# A late-surviving stem-ctenophore from the Late Devonian of Miguasha (Canada)

**DOI:** 10.1038/s41598-021-98362-5

**Published:** 2021-09-24

**Authors:** Christian Klug, Johanne Kerr, Michael S. Y. Lee, Richard Cloutier

**Affiliations:** 1 Paläontologisches Institut und Museum, Universität Zürich, Karl-Schmid-Strasse 4, 8006 Zurich, Switzerland; 2 Parc national de Miguasha, 231 Route de Miguasha Ouest, Nouvelle, QC G0C 2E0 Canada; 3 College of Science and Engineering, Flinders University, Adelaide, SA Australia; 4 Earth Sciences Section, South Australian Museum, Adelaide, SA Australia; 5 Département de Biologie, Chimie et Géographie, Université du Québec à Rimouski, 300 allée des Ursulines, Rimouski, QC G5L 3A1 Canada

**Keywords:** Evolution, Palaeontology, Phylogenetics, Taxonomy

## Abstract

Like other soft-bodied organisms, ctenophores (comb jellies) produce fossils only under exceptional taphonomic conditions. Here, we present the first record of a Late Devonian ctenophore from the Escuminac Formation from Miguasha in eastern Canada. Based on the 18-fold symmetry of this disc-shaped fossil, we assign it to the total-group Ctenophora. Our phylogenetic analyses suggest that the new taxon *Daihuoides jakobvintheri* gen. et sp. nov. falls near Cambrian stem ctenophores such as ‘dinomischids’ and 'scleroctenophorans'. Accordingly, *Daihuoides* is a Lazarus-taxon, which post-dates its older relatives by over 140 million years, and overlaps temporally with modern ctenophores, whose oldest representatives are known from the Early Devonian. Our analyses also indicate that the fossil record of ctenophores does not provide strong evidence for or against the phylogenomic hypothesis that ctenophores are sister to all other metazoans.

## Introduction

Palaeozoic sediments yield a growing number of fossil invertebrates with radial symmetries, some being quite enigmatic with body plans differing radically from those of extant organisms. Ctenophores (comb jellies) are one of the phylogenetically most important and controversial metazoan groups, and interest in their fossil record has been catalysed by new records of spectacularly preserved materials from Cambrian Lagerstätten from the 518-million-years-old Chengjiang Biota^1–8^, the 505-million-years-old Burgess Shale^9–11^ and other Burgess Shale-like deposits^12,13^.

Ctenophores have long been known to be near the base of Metazoa^14^, but some genetic and genomic studies have recently proposed that they are *the* most basal metazoans^15–21^, the 'ctenophores first' hypothesis. This has found some morphological support in a tentative Late Proterozoic stem-group representative of the ctenophores *sensu lato*^22^. Nevertheless, this phylogenetic hypothesis has been challenged; several molecular studies now favour the traditional morphologically-based arrangement where sponges are sister group to all other animals^23–28^.

Here, we present an invertebrate fossil from the Late Devonian Escuminac Formation (Miguasha, Quebec, Canada), a UNESCO world heritage site famous for its abundance of well-preserved vertebrate fossils including most major evolutionary groups of Devonian lower vertebrates from jawless fish to stem-tetrapods^29–38^. Based on morphological similarities of this Canadian fossil with stem-ctenophore fossils from the Cambrian Lagerstätte of the Chinese locality Chengjiang^7,39^, we assess its affinity to stem-group ctenophores (‘dinomischids’, *Siphusauctum*, ‘scleroctenophorans’^1,3,7,13^) and early crown group ctenophores. Modern ctenophores and many fossil forms lack mineralized hard parts, which renders the rare fossils that have been extracted from several Lagerstätten quite remarkable. Like the soft bodies of jellyfish and the polyps of hydrozoans and anthozoans, the probability for such soft bodies (or body regions) to become fossilized is extremely low. In spite of this low preservation potential, remains of stem-ctenophores have become known from several Cambrian and younger conservation deposits, and with even older candidate ctenophores in the Ediacaran^22^. While Cambrian Lagerstätten yielded several genera, ctenophore remains are much rarer in the Devonian; we are aware of only two studies, describing material from the German Hunsrück Slate^40,41^. This Early Devonian material, however, appears to belong to crown ctenophores morphologically similar to living forms such as *Pleurobrachia*, unlike the stem Cambrian taxa and the new Devonian stem taxon described here.

The most basal stem ctenophores are the ‘dinomischids’: sessile benthic petaloid invertebrates, many of which are equipped with a stalk. This group first was described from the Middle Cambrian Burgess Shale^9^. Based on the genus *Dinomischus*^9^, these early stalked forms were commonly called ‘dinomischids’. According to Zhao et al. (Ref^7^: 1113), dinomischids "form a grade in the lower part of the ctenophore stem group” and include taxa such as *Xianguangia*, *Daihua*, and *Dinomischus* that have hexaradiate-based symmetry (e.g., sixfold, 18-fold). Some later, skeletonised stem-ctenophores were termed ‘Scleroctenophora’ (Zhao et al.^7^: fig. 6; ‘scleroctenophorans’ have a shorter stalk, lack the ‘petals’ and have no bracts and might be monophyletic: Ou et al.^5^). To date, all known dinomischids and scleroctenophorans are Cambrian. Remarkably, analysis of the material described here suggests it is a very late-surviving member of this part of the ctenophore tree, occurring in strata over a hundred million years younger with no intervening known record, thus making it a Lazarus taxon with an extensive ghost lineage. The morphological similarities to Cambrian forms and the mix of characters regarding overall shape and symmetries render this discovery important. The aims of this study are to (1) describe the only known specimen of this Devonian ctenophore, (2) discuss its phylogenetic and systematic position, and the impact of fossil data for ctenophore affinities, and (3) assess its palaeoecological role.

## Results

### Geological setting

**Figure 1:**
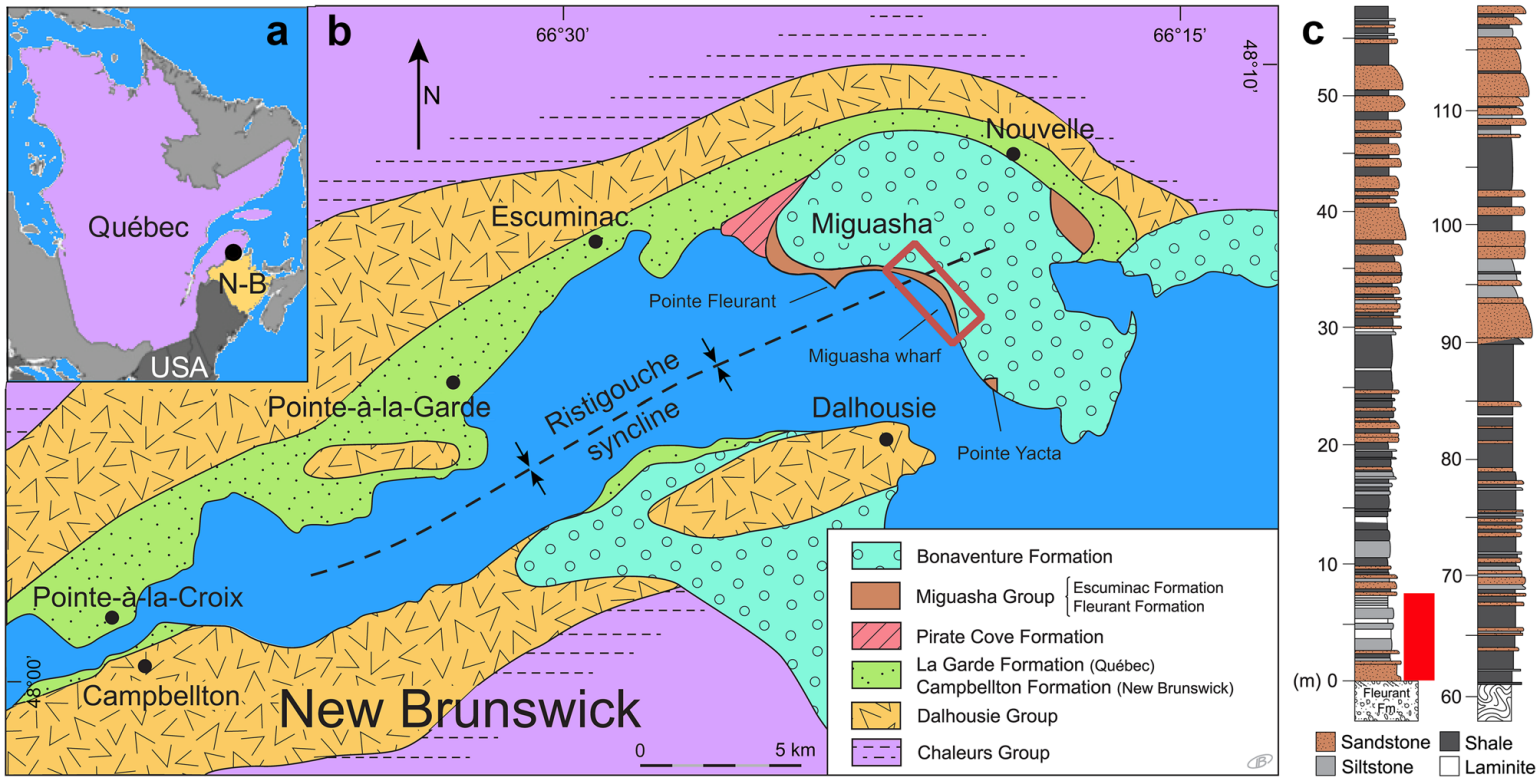
Geographic, geological and stratigraphic context of the Escuminac Formation. (**a**) Localisation of Miguasha in eastern Québec, Canada. (**b**) Geological map of the Escuminac Formation along the Ristigouche River, eastern Québec, Canada. (**c**) Simplified stratigraphic section of the Escuminac Formation showing the potential occurrence of *Daihuoides jakobvintheri* gen. et sp. nov. indicated by a red bar. Map and section modified from Cloutier et al.^29,30^.

The middle Frasnian^34^ (Late Devonian) Escuminac Formation, exposed on the south coast of the Gaspe Peninsula (eastern Quebec), is 119 m thick and forms steep cliffs up to 30 m high along the coast (Fig. 1). Five lithofacies are recognized^30^: rare coarse conglomerates (< 0.1% of total thickness), gray-green sandstones (25% of total thickness), carbonate-rich siltstones (22% of total thickness), shales (46% of total thickness), and thin shale-siltstone laminites (7% of total thickness). Konservat and Konzentrat Fossil-Lagerstätten horizons occur in the transgressive phase of the five sequences within an inner wave-dominated estuary^30^. The formation includes four depositional environments^30^: braided fluvial conglomerates, intertidal siltstones and argillites, bay head delta sandstones and siltstones, and central semi-enclosed basin shales.

### Taphonomy

**Figure 2:**
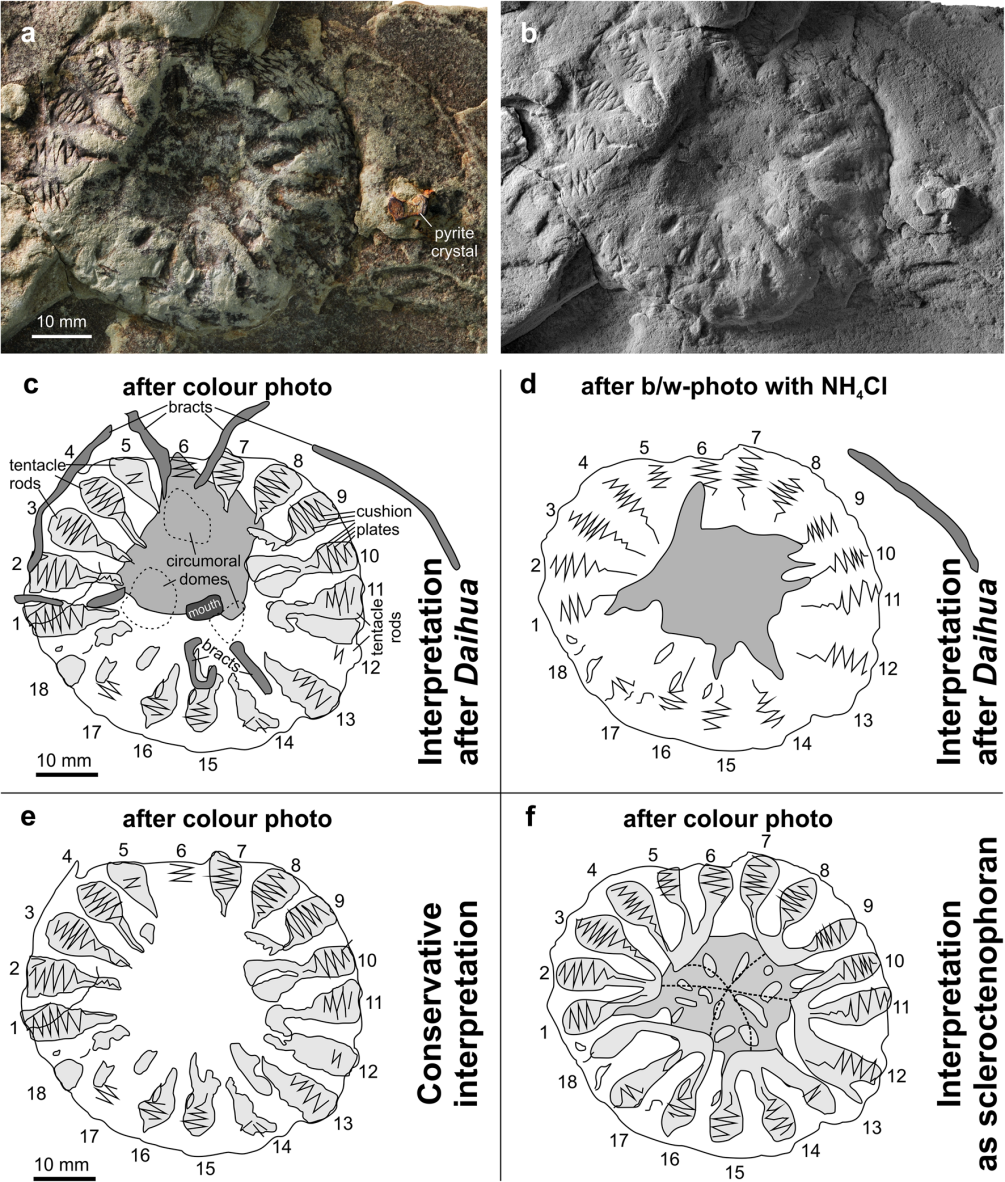
Photos and drawings of *Daihuoides jakobvintheri* n. gen. et sp. Holotype, MHNM 24-01, Escuminac Formation, Late Devonian, Miguasha. (**a**) color photo. (**b**) specimen coated with NH_4_Cl. (**c**–**f**) drawings after (**a**,**b**); light grey—tentacle rods, middle grey—oral surface, dark grey—bracts. Drawings of *D. jakobvintheri* n. gen. et sp. based on three alternative interpretations: (**c**, **d**) interpretation based on *Daihua*, (**e**) conservative interpretation, and (**f**) interpretation as a scleroctenophoran.

Specimen MHNM 24-01 is on the surface of a 3–4 cm thick, greyish-greenish fine siltstone bed with laminar, 1-cm thick graded beddings. Cubic pyrite crystals (Fig. 2a) and minute plant debris are present near the specimen. The specimen is only moderately deformed by sediment compaction. Anatomical details are preserved as elevations and furrows as well as hematite-stained surfaces. The precise stratigraphic position is unknown; the specimen was collected on the beach along the Miguasha cliff within the limits of the Miguasha National Park. Sedimentology and sediment colour suggest that the specimen came from the lower units of the Escuminac Formation.

Because of the gross similarity between jellyfish (Cnidaria) and comb jellies (Ctenophora), one might expect similar taphonomical conditions for their fossilisation. Young and Hagadorn^40^ reviewed the taphonomy of cnidarian medusae through their complete fossil record. Among the optimal preservation conditions, they reported the absence of bioturbation and rarity of scavengers, anoxic or hypoxic environment, rapid sediment burial, in sandy coasts, estuarine-lagoonal setting, mud-dominated open marine shelf. All these conditions are encountered for the occurrence of *Daihuoides* in fine-grained siltstone likely coming from the lower section of the Escuminac Formation.

### Faunal assemblage

In contrast to the early vertebrate diversity (20 species), the described invertebrate fauna (12 species) is relatively depauperate including only a few aquatic and continental taxa^29,30^. The aquatic component of the invertebrate fauna includes the abundant spinicaudatan *Asmusia membranacea*^32^ and extremely rare remains of a parastylonurid eurypterid^33^ as well as a scolecodont annelid^34^. The continental component, similarly rare, includes the millipede *Zanclodesmus willetti*^35^, the scorpion *Petaloscorpio bureaui*, and a gigantoscorpionid^33^. Fragments of arthropod cuticles, some of them likely referable to arachnids or trigonotarbids^30^, have been found in palynological preparations^34^. Two aquatic ichnotaxa (*Gyrophyllites* and *Planolites montanus*)^36,37^ have been identified, as well as two types of undescribed trace fossils^30^; ichnofossils and bioturbation are extremely rare thought the Escuminac Formation^29,30^. Until now, typical marine invertebrates were totally absent, a fact, which led earlier researchers^38^ to interpret the Miguasha palaeoenvironment as freshwater lakes. It is worth mentioning that we are not aware of any fully freshwater species of ctenophores, both living and fossil. Palynological^34^, geochemical^41,42^, sequential stratigraphy^30^ and vertebrate assemblage^43^ evidence suggest an estuarine setting with potential connectivity to a coastal marine environment.

### Systematic palaeontology

Metazoa

Total-group Ctenophora

**Comment:** In some characters (18 tentacle rods, alternating cushion plates), the only specimen of the new taxon resembles the Cambrian stem-ctenophore *Daihua* from the Early Cambrian of Chengjiang, China (Zhao et al.^7^: Fig. 2A). The 18-fold symmetry also occurs in other Cambrian genera (*Dinomischus* and *Xianguangia*^7^), thereby further supporting a stem position. Similarly, our phylogenetic analyses (based on the character matrix of Zhao et al.^7^) places *Daihuoides* gen. nov. on the ctenophoran stem, above the Cambrian dinomischids but below scleroctenophorans. The taxon is very different to the Early Devonian ctenophores from the Hunsrück Slate^44,45^, which are crown forms similar to living *Pleurobranchia*.

*Daihuoides* gen. nov.

**Type species**. *Daihuoides jakobvintheri* sp. nov.

**Etymology:** After *Daihua*, a stem-ctenophoran described by Zhao et al.^7^, referring to morphological similarities (thus the ending–*oides*) to the holotype of *D. sanqiong*, the only species of the genus *Daihua*.

**Diagnosis.** A large stem-group ctenophore with 18 radii carrying tentacles, which surround the oral surface/the central body axis. The calyx has a circular outline and carries 18 comb rows or tentacle rods with cushion plates arranged in a zigzag pattern. The comb rows or tentacle rods widen outward from the mouth and then taper again towards the edge of the calyx. Within this portion, the comb rows or tentacle rods carry ca. 10 larger, alternating cushion plates.

**Comments.** The only specimen shows only one end of the body , and due to taphonomic compression, it is uncertain whether this is the aboral (dorsal) or oral (ventral). This interpretation is further hampered by the possibility that the body might have collapsed, projecting the oral surface onto the aboral surface. In any case, several characters such as calyx shape, presence or absence of a stalk and morphological details of the tentacles are unknown. Additionally, the holotype of *Daihuoides jakobvintheri* gen. et sp. nov. is preserved in a fine-grained siltstone; thus, although some anatomical detail is discernible, the preservation differs profoundly from those of Cambrian occurrences, which are embedded in much finer grained claystones. Nevertheless, the overall similarity such as the presence of 18 tentacle rods (Fig. 2) and possibly three oral domes are here considered adequate to include near stem-group ctenophores (e.g., ‘dinomischids’) sensu Zhao et al.^7^.

We name the Devonian genus *Daihuoides* after the Cambrian genus *Daihua* referring to its similarity in the arrangement of structures resembling tentacle rods (Zhao et al.^7^: Fig. 2A) and the zigzag appearance of the possible cushion plates (Zhao et al.^7^: Fig. 2E). Among all the illustrations of Cambrian ‘dinomischids’, it is *Daihua*, which shows the greatest degree of resemblance enabling the homologisation of several organs.

The only other stem ctenophore showing a high degree of resemblance is *Ctenorhabdotus*. This genus lacks a stalk. It has a subspherical calyx with eight partitions on the surface that branch into three comb rows each (Conway Morris and Collins^10^: figs. 23, 24). Accordingly, there are 24 rows, six more than in *Daihuoides* gen. nov. However, depending how one interprets the visible structures in the Escuminac specimen, one could infer a grouping into three comb rows (Fig. 2f). Notably, both 18 and 24 represent multiples of planes of symmetries commonly occurring in ctenophorans and cnidarians, namely four and six.

The new taxon differs from all Palaeozoic taxa in the alternating arrangement of comb rows or cushion plates. Also, the short tentacle rods or comb rows do not resemble the conditions seen in Cambrian stem-ctenophores. The unique combination of characters, and its huge stratigraphic separation from similar taxa (Cambrian), justifies placing it in a new genus.

*Daihuoides jakobvintheri* sp. nov.

**Etymology**. Honouring Jakob Vinther (University of Bristol) for his substantial contributions on soft-bodied organisms from the Early Palaeozoic, which are still understudied.

**Holotype**. MHNM 24-01.

**Nomenclatural statement.** A Life Science Identifier (LSID) was obtained for the new genus and species (*Daihuoides jakobvintheri*): urn:lsid:zoobank.org:act: D3DF39BB-5794-4842-A833-655DD72A1F76, and for this publication: urn:lsid:zoobank.org: pub: 93A203AA-B0EA-42A2-8295-AD07B85E1359.

**Material**: Holotype specimen MHNM 24-01.

**Locality, horizon and age.** Miguasha, Quebec, Canada; Escuminac Formation, middle Frasnian, Devonian.

**Diagnosis**. Same as genus definition, due to monotypy.

**Description**. Depending on the morphological and taxonomic interpretation, the holotype MHNM 24-01 is exposed from the oral or the aboral side. It is disc-shaped with a roughly circular outline; the calyx has a diameter of 58.2 mm. It carries a circular bulge that is 17.8 mm wide, surrounding a central depression of approximately 20 mm diameter. This depression is largely stained by iron oxides, which cover an irregular surface of about 28 mm in diameter (middle grey in Fig. 2c). This surface has some roughly radially arranged projections giving it a star-shaped outline. Micro-CT scan analysis did not reveal any internal anatomical detail.

**Figure 3:**
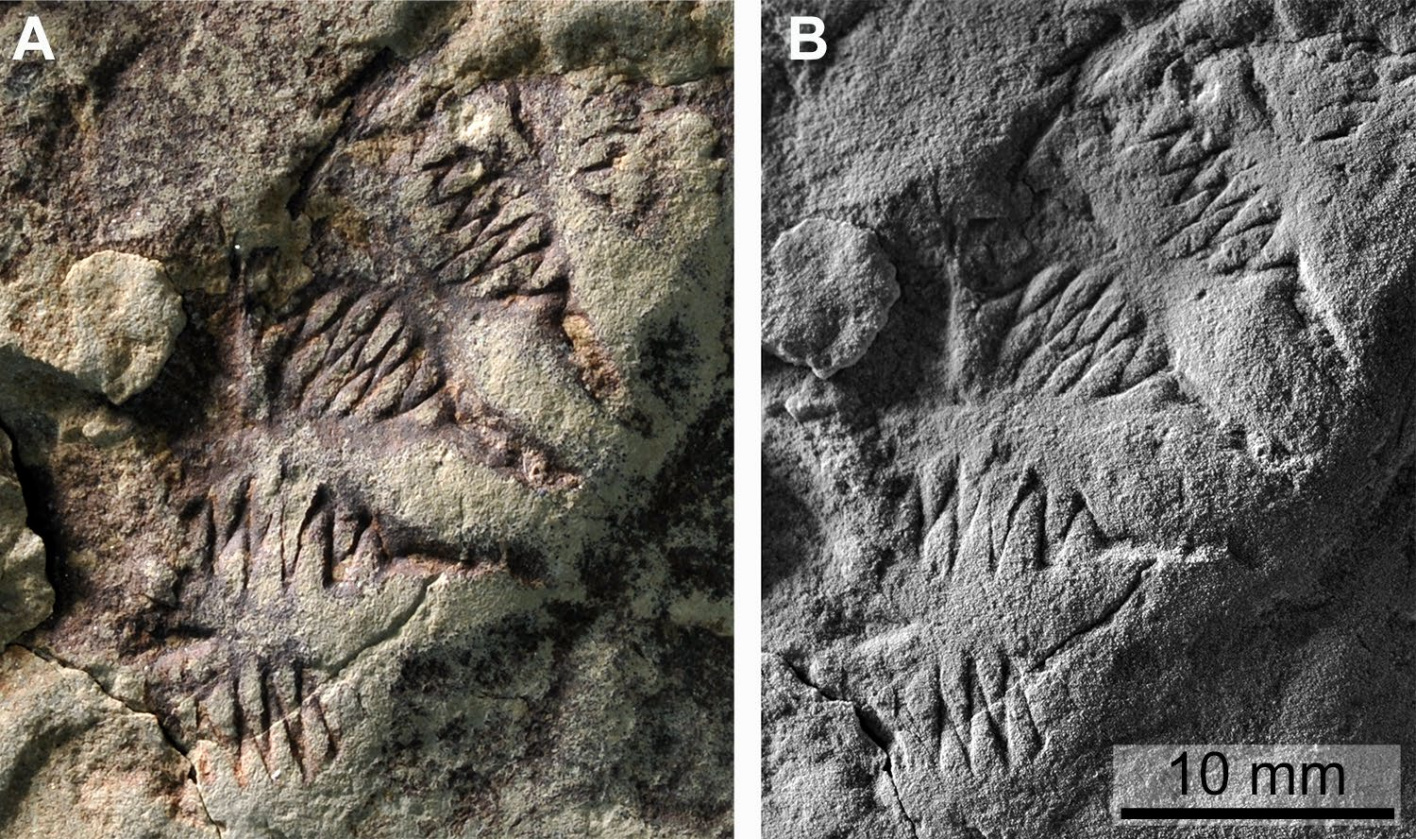
Morphological details of *Daihuoides jakobvintheri* n. gen. et sp. Holotype, MHNM 24-01, Escuminac Formation, Late Devonian, Miguasha. (**A**) color photo showing the tentacle rods/ cushion plates. (**B**) same as in (**A**) but specimen coated with NH_4_Cl.

The bulge surrounding this depression carries 18 club-shaped fields that are arranged radially. Although these fields are quite strongly eroded in some places, all show remains of deeply incised, haematite-stained zigzag-lines (Fig. 2). The amplitude of the single bends reaches 7.6 mm in the broadest part of the field and becomes strongly reduced towards the centre of the oral surface (Fig. 3). In the broader part of the club-shaped fields (‘tentacle rods’ or ‘comb fields’?), about ten such bends can be seen. The single fields alternate and the zigzags are pointed in both directions. The narrow furrow first reduces the intensity of its curvature and then fades out inward. The club-shaped field is slightly longer than the furrow. Additional furrows and impressions are somewhat irregular and two alternative interpretations are discussed below.

## Discussion

The holotype of *Daihua sanqiong* shows 18 elongate tentacle rods, the elongate tips of which protrude far beyond the disc-shaped body. In addition, the oral surface carries three circumoral domes, which, in typical ctenophores, give rise to a pair of long tentacles termed bracts by Zhao et al.^7^. The number of ‘tentacle rods’ and the arrangement of cushion plates on them is similar in *Daihuoides* gen. nov., but the oral surface is quite irregular and unequivocal interpretations are impossible without better preserved material. With only 18 rays, any symmetry in the central structure must have been hexameral or triradiate. Perhaps this surface encompassed the three oral domes (dashed lines in Fig. 2c), which would measure about 10 mm in width, if that interpretation is correct. In turn, this interpretation is linked with the presence of 1–2 mm wide furrows (dark grey in Fig. 2c), partially filled with iron oxides, which extend from the tentative circumoral domes; accordingly, these furrows might represent remains of the bracts. Another tentative bract extends outside of the calyx (top right in Fig. 2a–d). A depression measuring 4 times 6 mm is interpreted with reservation as mouth according to its position between the supposed circumoral domes.

**Figure 4:**
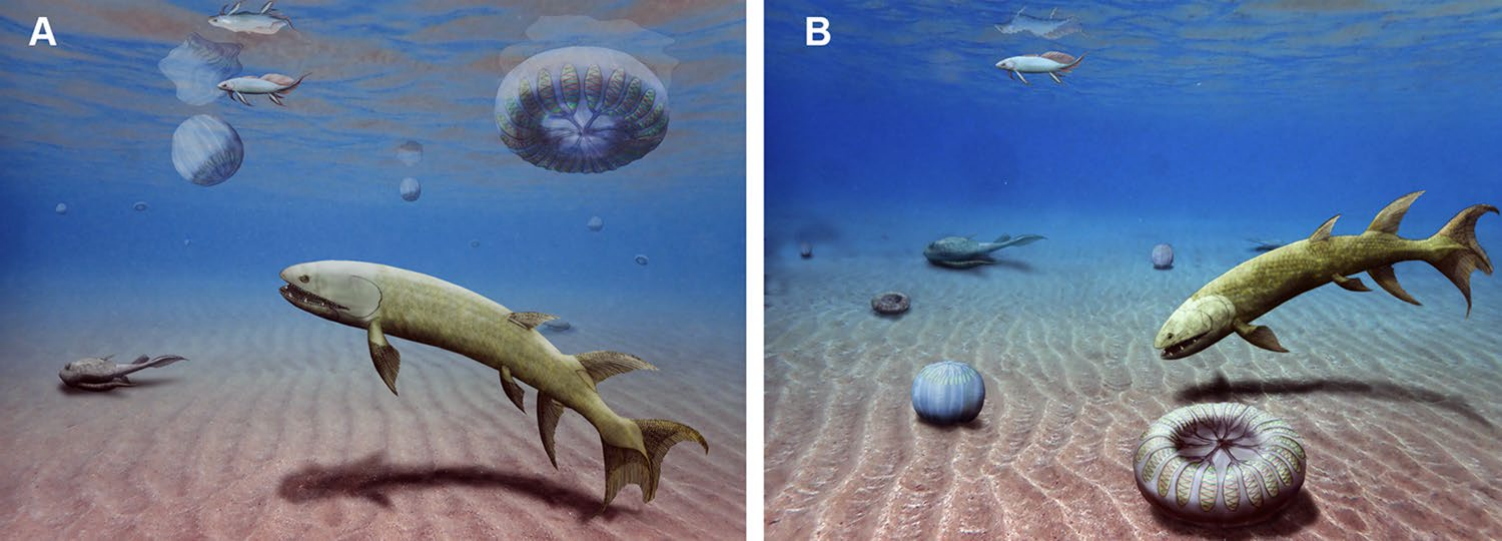
Reconstructions. Corresponding to its transitional position, we interpreted *Daihuoides jakobvintheri* n. gen. et sp. from the Devonian of Canada as a planktonic animal like many crown group comb jellies (**A**) or as benthic organism like ‘dinomischids’ (**B**). Note the alternative interpretations of the body shape as being either more disc-shaped or more spherical.

Although the Burgess Shale form *Ctenorhabdotus*^10^ differs from *Daihuoides* gen. nov. in possessing 24 versus 18 radii, they share tentacles that do not extend beyond the disc, and similar connections between comb rows^10^. Some specimens of *Ctenorhabdotus* display a central structure showing the tetrameral symmetry; the four initial rays branch again into eight, which then lead to three comb-rows each. We interpreted the fossil accordingly in Fig. 2f, but these connections between comb rows are arguable (Figs. 2c–e, 3). When comparing *Daihuoides* gen. nov. to published specimens of *Ctenorhabdotus*^7^, it is also conceivable that we see the comb rows arranged in groups of three around the apical organ. In Fig. 4, we show alternative reconstructions as benthic and planktic forms with spherical to flattened circular body form.

**Figure 5:**
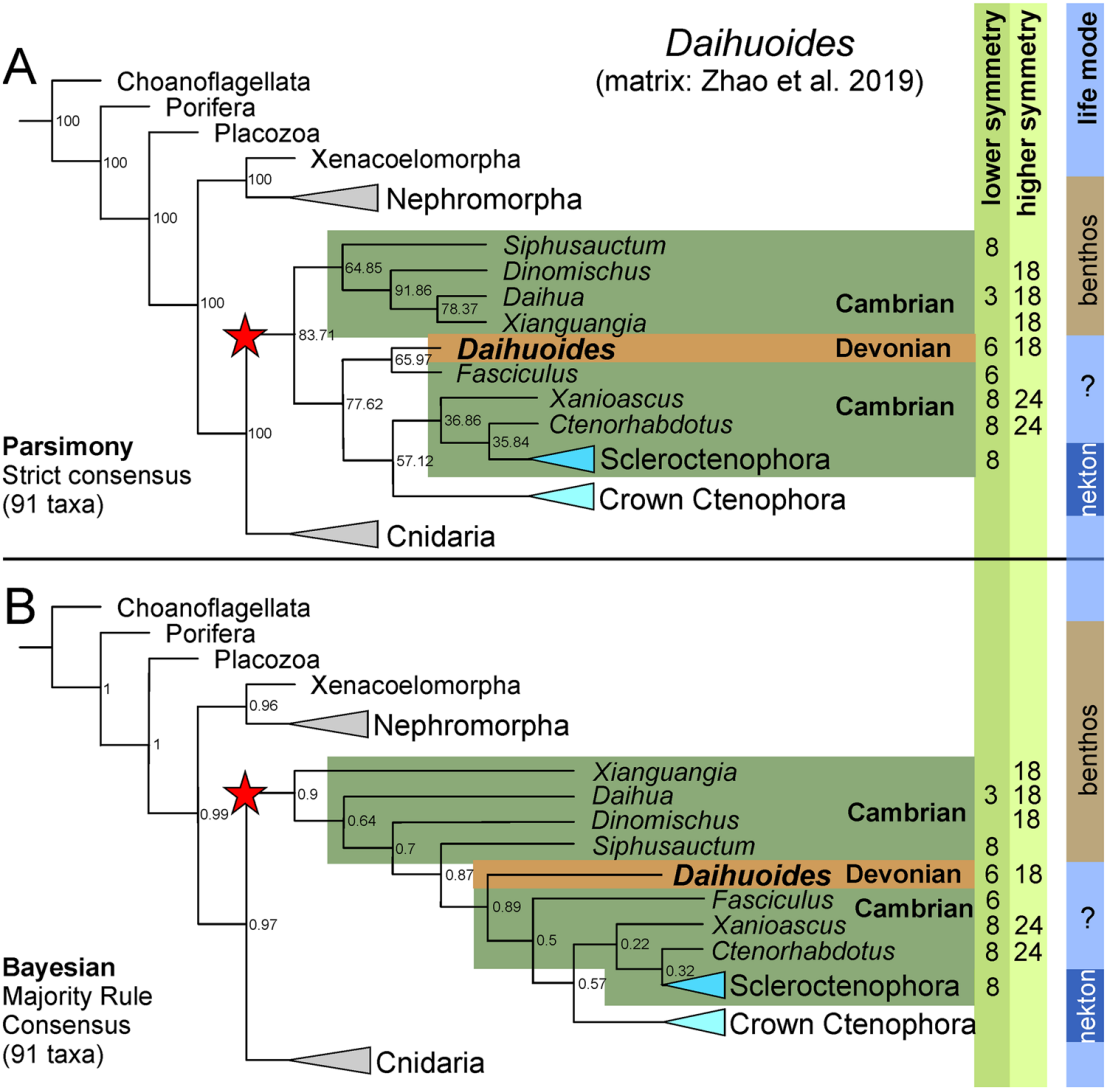
Phylogenies. Possible phylogenetic positions of *Daihuoides* based on data matrix derived from Zhao et al.^7^. For the character matrix and complete trees showing all taxa and details see the Supplementary Information. Note the distribution of higher and lower symmetries (light green fields) as well as the mode of life on the right (blue/brown fields). (**A**) Simplified strict consensus tree from parsimony analysis; numbers at nodes denote bootstrap percentages. (**B**) Simplified majority-rule consensus tree from Bayesian inference, numbers at nodes denote posterior probabilities. Star = total-group Ctenophora. Dark green colour on tree = Cambrian taxa. Brown colour on tree = Devonian (here only *Daihuoides*).

We performed parsimony and Bayesian phylogenetic analyses using a character-by-taxon matrix based on Zhao et al.^7^ (“Methods” section). The consensus trees of these analyses places *Daihuoides* gen. nov. mid-way along the stem leading to crown-group Ctenophora (Fig. 5; see Figs. S1–S4). Its inclusion in the crown-group appears unlikely because of its symmetry and its benthic habitat (most modern taxa are planktic; the only recent benthic group, the Platyctenida, has a quite different morphology and usually measures less than 10 mm^46^. In an earlier study by Ou et al. (Ou et al.^5^: fig. 4), the living *Beroida* is the sister taxon to all other (fossil and recent) ctenophores, thereby placing all fossil forms in the crown-group. However, Zhao et al.^7^ retrieved a much-reduced crown-group, and this arrangement is replicated in our study (as would be expected). However, contrary to Zhao et al.^7^, the fossil evidence does not increase support for a ctenophore-cnidarian (coelenterate) clade: this hypothesis remains supported by four or five steps over the ctenophores-first hypothesis, whether fossils are included or excluded (Methods).

*Daihuoides* gen. nov. appears to be a transitional taxon between the most basal stem ctenophores (the benthic ‘dinomischids’), and higher stem ctenophores (such as scleroctenophorans, where stalks were reduced)*.* It has the hexaradial-based symmetry of dinomischids, rather than the tetraradial-based symmetry of more crownward ctenophores. However, it resembles crownward stem ctenophores such as *Ctenorhabdotus* in lacking distinct tentacles around a crown. It thus falls right in the middle of the ctenophore stem. Notably, however, all stem taxa immediately above and below *Daihuoides* gen. nov. are Cambrian: *Daihuoides* gen. nov. is thus more than 100 million years younger than all other taxa in that region of the tree. In turn, this implies that Ordovician and Silurian conservation deposits will sooner or later yield comparable stem ctenophorans, which will help to improve our understanding of the evolution of these early forms.

The most basal ctenophores (‘dinomischids’) were stalked benthic forms, while more crownward ctenophores have reduced stalks and are often entirely pelagic. The position of *Daihuoides* gen. nov. near the boundary of these two grades helps fill in a major gap in ctenophore evolution. The Devonian age of this fossil also provides important temporal continuity between these Cambrian stem-ctenophores and living Ctenophores. Notably, the fossil record of modern ctenophores extends at least to the Early Devonian, with forms very similar to living *Pleurobrachia* preserved in the Hunsrück slate (Pragian-Emsian). The presence of *Daihuoides* gen. nov. in the Late Devonian implies temporal overlap of Cambrian-type stem-ctenophores and modern ctenophores across much of the Devonian.

Accepting the position of *Daihuoides* gen. nov. as a late-surviving dinomischid, the Ctenophora exhibit a coherent picture of an evolution from sessile to planktic forms with a change in symmetry from a high number of radii (18 or 24) to a low multiple of 2 (4 or 8). The take-off from sediment is a recurrent feature in many clades happening in the early Palaeozoic. Living ctenophores are also predominantly marine, occasionally entering brackish habitats^47^. Brackish-marine salinities as likely prevailed in Miguasha^41,42^, raising the possibility that *Daihuoides* was also a brackish water taxon. However, it appears more plausible that this ctenophore was transported from the open sea into an estuary, which would also explain its scarcity; the presence of a scolecodont and acritarchs found in the lower section of the Escuminac Formation also suggested this marine connectivity^30,34^.

## Methods

The specimen was photographed with a Nikon D-300 with an objective AF-S micro Nikkor 60 mm under white light both in colour and in black and white after it was coated with NH_4_Cl-sublimate. It was scanned with a SkyScan using the following parameters: 130 kV, 61 μA, 0.25 mm brass filter, 5000 ms exposure time, 3684 projections with an angular step of 0.2°, voxel resolution of 35.54 µm. The 3D reconstruction was made using NRecon (Bruker micro CT, 2016) and generated Tiff (16 bytes) images. Drishti Import and Drishti 2.6.4 and 2.6.5 (beta version) were used for manual segmentation of the specimen^45,48^.

We carried out phylogenetic analyses using by adding the new taxon, and one new character, to the character-by-taxon matrix of Zhao et al.^7^, resulting in a matrix of 94 taxa and 279 characters. The codings for the new taxon and character states are available in Appendix 1, and the full matrices are available as executables (see below).

We performed both phylogenetic analyses using both parsimony and Bayesian methods. Following Zhao et al.^7^, all characters were treated as unordered, Choanoflagellata were the outgroup, and analyses used the full matrix (94 taxa), as well as a reduced matrix (91 taxa, 3 contentious Ediacaran taxa deleted).

Parsimony analyses used PAUP* 4.0a168^49^, the following settings were used to ensure all tree islands were sampled, but large islands did not clog up all memory: <HSEARCH addseq = random nreps = 500 rstatus = yes nchuck = 1000 chuckscore = 1 enforce = no;>. Executable files with all these settings (and other relevant settings) are in SI Appendix 2. The strict and majority-rule consensus trees for the 94 taxon and 91 taxon analyses are shown in Figs. S1–S2. Node support was assessed with 200 nonparametric bootstrap replicates, and these values are shown in Fig. S3.

Bayesian analyses used MrBayes 3.2.7^50^, with rate variability across characters was accommodated using a gamma parameter. Each analysis used 4 runs (each with 4 chains and heating temperature of 0.1), with 20 million generations and a burning of 0.25. Stationarity was confirmed across runs with PSRF close to 1 and standard deviations of split (clade) frequencies very close to 0. Executable files are in SI Appendix 3. The majority-rule consensus trees are shown in Fig. S4.

To evaluate whether the addition of fossil ctenophores support for the Ctenophoran-Cnidarian clade, we compared the lengths of the best unconstrained trees (which grouped ctenophorans and cnidarians), and the best trees which placed ctenophorans as basal to all other metazoans as per genomic studies. These competing topologies were compared using data matrices which included only living taxa, and living taxa + fossils (this approach was performed using both the unmodified Zhao et al.’s^7^ matrix, and the present matrix). Using the Zhao et al.’s matrix, when only 77 extant taxa were considered, the basal position of ctenophorans entailed an extra 4 steps (382 vs. 378 steps). When all fossil taxa were included (93 taxa), the basal position of ctenophorans entailed 5 steps (426 vs. 421); when the 3 contentious fossil forms were excluded (90 taxa), this changed to 4 steps (421 vs. 417). We repeated this approach with our slightly expanded matrix, with identical results: with 77 extant taxa, the difference was 4 steps (385 vs. 381), with all 94 living and fossil taxa, it was 5 steps (437 vs. 432), and with 91 living and non-contentious fossil taxa, the difference was 4 steps (431 vs. 427). Executable files for topology tests are in Appendix 4.

The fossil record therefore does not increase support for ctenophores having cnidarian affinities, relative to an alternative basal metazoan position. The difference between these hypotheses remains at 4 or 5 steps, whether fossils are excluded or included.

## Supplementary Information

Supplementary Information 1.

Supplementary Information 2.

Supplementary Information 3.

Supplementary Information 4.

Supplementary Information 5.

Supplementary Information 6.

Supplementary Information 7.

Supplementary Information 8.

Supplementary Information 9.

Supplementary Information 10.

Supplementary Information 11.

Supplementary Information 12.

Supplementary Information 13.

Supplementary Information 14.

Supplementary Information 15.

Supplementary Figures.

## Data Availability

Specimen MHNM 24-01 is curated in the collections of the Musée d’Histoire naturelle de Miguasha (MHNM), parc national de Miguasha. Data sets generated during the current study, including raw tiff stacks from µCT analyses are available from the corresponding authors on reasonable request. Data matrices, list of characters, and PAUP/MrBayes executables and trees are available as supplementary information (Appendices 1–4 and Figs. S1–S4).

## References

[1] Chen J-Y, Hou X-G, Lu H-Z (1989). Early Cambrian hock glass-like rare sea animal *Dinomischus* (Entoprocta) and its ecological features. Acta Palaeontol. Sin..

[2] Chen J-Y (2007). Raman spectra of a lower Cambrian ctenophore embryo from southwestern Shaanxi, China. Proc. Natl. Acad. Sci. U.S.A..

[3] Peng J, Zhao Y-L, Lin JP (2006). *Dinomischus* from the middle Cambrian Kaili Biota, Guizhou, China. Acta Geol. Sin..

[4] Han J (2010). Tiny sea anemone from the Lower Cambrian of China. PLoS ONE.

[5] Ou Q (2015). A vanished history of skeletonization in Cambrian comb jellies. Sci. Adv..

[6] Ou Q (2017). Three Cambrian fossils assembled into an extinct body plan of cnidarian affinity. Proc. Natl. Acad. Sci. U.S.A..

[7] Zhao Y (2019). Cambrian sessile, suspension feeding stem-group ctenophores and evolution of the comb jelly body plan. Curr. Biol..

[8] Daley AC, Antcliffe JB (2019). Evolution: The battle of the first animals. Curr. Biol..

[9] Conway Morris S (1977). A new entoproct-like organism from the Burgess Shale of British Columbia. Palaeontology.

[10] Conway Morris S, Collins D (1996). Middle Cambrian ctenophores from the Stephen Formation, British Columbia. Canada. Philos. Trans. R. Soc. Lond. B Biol. Sci..

[11] O’Brien LJ, Caron J-B (2012). A new stalked filter-feeder from the middle Cambrian Burgess Shale, British Columbia, Canada. PLoS ONE.

[12] Mikulas R, Kordule V (1998). A problematic fossil from the Middle Cambrian of the Barrandian area (Czech Republic). J. Geosci. (Prague).

[13] Kimmig J, Strotz LC, Lieberman BS (2017). The stalked filter feeder *Siphusauctum lloydguntheri* n. sp. from the middle Cambrian (Series 3, Stage 5) Spence Shale of Utah: Its biological affinities and taphonomy. J. Paleontol..

[14] Wallberg A, Thollesson M, Farris JS, Jondelius U (2004). The phylogenetic position of the comb jellies (Ctenophora) and the importance of taxonomic sampling. Cladistics.

[15] Dunn CW (2008). Broad phylogenomic sampling improves resolution of the animal tree of life. Nature.

[16] Philippe H (2009). Phylogenomics revives traditional views on deep animal relationships. Curr. Biol..

[17] Rokas A (2013). My oldest sister is a sea walnut?. Science.

[18] Ryan JF (2013). The genome of the ctenophore *Mnemiopsis leidyi* and its implications for cell type evolution. Science.

[19] Moroz LL (2014). The ctenophore genome and the evolutionary origins of neural systems. Nature.

[20] Halanych KM, Whelan NV, Kocot KM, Kohn AB, Moroz LL (2016). Miscues misplace sponges. Proc. Natl. Acad. Sci. U.S.A..

[21] Halanych KM (2015). The ctenophore lineage is older than sponges? That cannot be right! Or can it?. J. Exp. Biol..

[22] Tang F, Bengtson S, Wang Y, Wang XL, Yin CY (2011). *Eoandromeda* and the origin of Ctenophora. Evol. Dev..

[23] Pick KS (2010). Improved phylogenomic taxon sampling noticeably affects nonbilaterian relationships. Mol. Biol. Evol..

[24] Pisani D (2015). Genomic data do not support comb jellies as the sister group to all other animals. Proc. Natl. Acad. Sci. U.S.A..

[25] Pisani D (2016). Reply to Halanych et al.: Ctenophore misplacement is corroborated by independent datasets. Proc. Natl. Acad. Sci. U.S.A..

[26] Feuda R (2017). Improved modeling of compositional heterogeneity supports sponges as sister to all other animals. Curr. Biol..

[27] Simion P (2017). A large and consistent phylogenomic dataset supports sponges as the sister group to all other animals. Curr. Biol..

[28] Kapli P, Telford MJ (2020). Topology-dependent asymmetry in systematic errors affects phylogenetic placement of Ctenophora and Xenacoelomorpha. Sci. Adv..

[29] Cloutier R (2013). Great Canadian Lagerstätten 4. The Devonian Miguasha biota (Québec): UNESCO World heritage site and a time capsule in the early history of vertebrates. Geosci. Can..

[30] Cloutier R, Proust J-N, Tessier B (2011). The Miguasha Fossil-Fish-Lagerstätte: A consequence of the Devonian land–sea interactions. Palaeobiodivers. Palaeoenviron..

[31] Cloutier R (2020). *Elpistostege* and the origin of the vertebrate hand. Nature.

[32] Martens T, Schultze H-P, Cloutier R (1996). Devonian Fishes and Plants of Miguasha, Quebec, Canada.

[33] Jeram AJ, Schultze H-P, Cloutier R (1996). Devonian Fishes and Plants of Miguasha, Quebec, Canada.

[34] Cloutier R, Loboziak S, Candilier A-M, Blieck A (1996). Biostratigraphy of the Upper Devonian Escuminac Formation, eastern Quebec, Canada: A comparative study based on miospores and fishes. Rev. Palaeobot. Palynol..

[35] Wilson HM, Daeschler EB, Desbiens S (2005). New flat-backed archipolypodan millipedes from the Upper Devonian of North America. J. Paleontol..

[36] Maples C, Schultze H-P, Cloutier R (1996). G. Devonian Fishes and Plants of Miguasha, Québec, Canada.

[37] Schultze H-P, Horn MH, Martin KLM, Chotkowski MA (1999). Intertidal Fishes: Life in Two Worlds.

[38] Dineley DL, Williams BPF, Klein GV (2021). Symium—Continental Sedimentation in Northeastern North America.

[39] Chen J-Y, Erdtmann B-D, Simonetta AM, Conway Morris S (1991). The Early Evolution of Metazoa and the Significance of Problematic Taxa.

[40] Young GA, Hagadorn JW (2020). Evolving preservation and facies distribution of fossil jellyfish: a slowly closing taphonomic window. Boll. Soc. Paleontol. Ital..

[41] Schmitz B, Åberg G, Werdelin L, Forey P, Bendix-Almgreen SE (1991). ^87^Sr/^86^Sr, Na, F, Sr, and La in skeletal fish debris as a measure of the paleosalinity of fossil-fish habitats. Geol. Soc. Am. Bull..

[42] Matton O, Cloutier R, Stevenson R (2012). Apatite for destruction: Isotopic and geochemical analyses of bioapatites and sediments from the Upper Devonian Escuminac Formation (Migiasha, Québec). Palaeogeogr. Palaeoclimatol. Palaeoecol..

[43] Schultze H-P, Cloutier R (1996). Devonian Fishes and Plants of Miguasha.

[44] Stanley GD, Stürmer W (1983). The first fossil ctenophore from the lower devonian of West Germany. Nature.

[45] Stanley GD, Stürmer W (1987). A new fossil ctenophore discovered by X-rays. Nature.

[46] Glynn PW, Coffman B, Primov KD, Moorhead SG, Vanderwoude J, Barrales RN, Williams MK, Roemer RP (2018). Benthic ctenophores (Platyctenida: Coeloplanidae) in South Florida: Predator–prey interactions. Inverteb. Biol..

[47] Costello JH, Bayha KM, Mianzan HW, Shiganova TA, Purcell JE (2012). Transitions of *Mnemiopsis leidyi* (Ctenophora: Lobata) from a native to an exotic species: A review. Hydrobiologia.

[48] Limaye, A. in *Proceedings of SPIE—Developments in X-Ray Tomography VIII.* (ed S.R. Stock).

[49] Swofford DL (2003). PAUP*: Phylogenetic Analysis Using Parsimony (*and other methods_). Version 4.

[50] Ronquist F, Teslenko M, Van Der Mark P, Ayres DL, Darling A, Höhna S (2012). MrBayes 3.2: efficient Bayesian phylogenetic inference and model choice across a large model space. System. Biol..

