# Supplementary material for: A late-surviving stem-ctenophore from the Late Devonian of Miguasha (Canada)
Source: Sci Rep. 2021 Sep 24;11:19039. doi: 10.1038/s41598-021-98362-5 (PMC8463547; doi:10.1038/s41598-021-98362-5)
Supplement: Supplementary file 1 — Supplementary Information 1. [file 41598_2021_98362_MOESM1_ESM.docx]

Character-by-Taxon Matrix

The first 278 characters follow Zhao *et al*. (2016).

Character 279 is: Lower (primary) symmetry:

state 0 = 2-fold

state 1 = 3-fold

state 2 = 4-fold
state 3 = 6-fold
state 4 = 8-fold

Codings for *Daihuoides* for all 279 characters are as follows (inapplicables, in the executable matrix as dashes, are here denoted by N to avoid confusion with hyphenated line breaks).

Daihuoides

??????????????????????????????00000?0???0????00000N???0?0000??0?????(01)

???????11000???????110?????0?0???????????N?????????0????????NNN0??????????????????????????0NNNNN?????????????00???????????????N????????????N?????????????????????????????????????????????????????????????????????3

The full matrix in executable Nexus formats (for PAUP and MrBayes) are available as SI files.

**Reference:**

Zhao, Y. *et al.* Cambrian sessile, suspension feeding stem-group ctenophores and evolution of the comb jelly body plan. *Current Biology* **29**, 1112–1125 (2019).
