# Supplementary material for: A late-surviving stem-ctenophore from the Late Devonian of Miguasha (Canada)
Source: Sci Rep. 2021 Sep 24;11:19039. doi: 10.1038/s41598-021-98362-5 (PMC8463547; doi:10.1038/s41598-021-98362-5)
Supplement: Supplementary file 14 — Supplementary Information 14. [file 41598_2021_98362_MOESM14_ESM.doc]

#NEXUS

BEGIN DATA;

DIMENSIONS ntax=94 nchar=279;

FORMAT DATATYPE=standard MISSING=? GAP=- INTERLEAVE=YES SYMBOLS = "0 1 2 3 4 5";

MATRIX

Choanoflagellata 10-------00--0-0--0--------------------------0--------------------00-----00----------0----------------0000-----0-0-?---00------0----0---0--00-0----------------0--------?--0-----0---?---------------------------------------------0--------------------??--------------------0-0--0---

Porifera 11(0 1)00101(0 1)10--0000-0000---0----00000-0--000-0000000-0---000000-----00----00000--------0---------------?0000-----0-0-0---00----000----00-101100-(0 1)00--------------0--------?--0-----0---?---------------------------------------------0--------------------??--------------------0-0--0---

Placozoa 01?00101010--1000-1000---0-0--00000-0--000-0000000-0---000000-----00----00000--------0---------00----?0000---0-0-0-0---00----000----0--?1000--000--------------0--------?--0-----0---?---------------------------------------------0--------------------??--------------------0-0--0---

Xianguangia ????????????????????01?0-?????00000?0??0000??00000-???0?0000??0????????-????1011?10-01000100-10?0-0???????????-?????????0----??1100000-?10?0--?1?1?????????????0-----??0?????????100011????????????0?0?????0??1?4???????????0?????????????????????????????????????????????????????????3

Daihua ?????????????????????1?0-?????00000?0??00?0??00000-???0?0000??0?????0??-????1011111001000100-11?0-0????????1????????????0??????1100000-?10?0???1???????????????0-----??0?????????100011????????????0?0?????0??1?4???????????0?????????????????????????????????????????????????????????1

'Dinomischus_venustus'????????????????????01?0-?????00000?0??00?0??00000-???0?0000??0????????-????1011111001000110-11?000???????????-?????????0----??1?00000-?10?0???0???????????????0-----??0?????????100011????????????0?0?????0??1?4???????????0??????????????????????????????????????????????????????????

Siphusauctum ????????????????????01?0-?????00000?0??00?0??00000-???0?0000??0?????5??-????1110111001000210-01?000????????1??-?????????0??????0----10-?10?0???0???????????????0-----??0?????????100000????????????0?0?????0??1?0???????????0?????????????????????????????????????????????????????????3

Namacalathus ???????????????????????????????????????????????????????????0????????5??????????????????????0-?1????????????????????????????????1??00???????????????????????????0-----????????????1??????????????????????????????????????????0?????????????????????????????????????????????????????????3

Eolympia ?????????????????????1?0??????00?0??0????????00????????????0????????0???????100???????????00-?1??-??????????????????????0?---??????????????????1???????????????0-----????????????100011??????0?????0?0????????1?4???????????0?????????????????????????????????????????????????????????4

Daihuoides ??????????????????????????????00000?0???0????00000-???0?0000??0?????(0 1)???????11000???????110?????0?0???????????-?????????0????????---0??????????????????????????0-----?????????????00???????????????-????????????-?????????????????????????????????????????????????????????????????????3

Fasciculus ????????????????????0?????????00000?0??00????00000-???0?0000??0?????1??-????11000?11???1141??00?0?0????????1??-?????????0----??0----00-??????????-?????????????0-----??????????????????????????????-????????????-???????????0?????????????????????????????????????????????????????????3

Xanioascus ????????????????????0?????????00000?0??00????00000-???0?0000??0?????1??-????11000?11???10311000?0?0??????1????-?????????0----??0----00-??????????-?????????????0-----??????????????????????????????-????????????-???????????0?????????????????????????????????????????????????????????4

Ctenorhabdotus ????????????????????0?????????00000?0??00????00000-???0?0000??0?????1??-????11000?11???11311000?0?0???????????-?????????0----??00---010??????????-?????????????0-----?????????????00???????????????-????????????-???????????0?????????????????????????????????????????????????????????4

Gemmactena ??????????????????????????????00000?0??00????00000-???0?0000??0????????-????11000?1?????00111?0?0?0????????1??-?????????0----??10110111??????????-?????????????0-----??????????????????????????????-????????????-???????????0?????????????????????????????????????????????????????????4

Thaumactena ????????????????????0?????????00000?0??00????00000-???0?0000??0????????-????11000?10????00111?0?0?0???????????-?????????0----??10?00110??????????-?????????????0-----??????????????????????????????-????????????-???????????0?????????????????????????????????????????????????????????4

Galeactena ????????????????????0?????????00000?0??00????00000-???0?0000??0????????-????11000?10????00111?0?0?0???????????-?????????0----??10?00110??????????-?????????????0-----??????????????????????????????-????????????-???????????0?????????????????????????????????????????????????????????4

Batofasciculus ??????????????????????????????00000?0??00????00000-???0?0000??0????????-????1100???0???100111?0?0?0???????????-?????????0----??10101111??????????-?????????????0-----??????????????????????????????-????????????-???????????0?????????????????????????????????????????????????????????4

Maotianoascus ????????????????????0?????????00000?0??00????00000-???0?0000??0????????-????11000?10????00110?0?0?0????????1??-?????????0----??10???110??????????-?????????????0-----??????????????????????????????-????????????-???????????0?????????????????????????????????????????????????????????4

Trigoides ??????????????????????????????00000?0??00????00000-???0?0000??0????????-????1100??1?????00111?0?0?0???????????-?????????0----??10110111??????????-?????????????0-----??????????????????????????????-????????????-???????????0?????????????????????????????????????????????????????????4

Cydippida 0100111111111000101011-100-0-000000-00000010000000-0--0000000000000010011110110001111111001100000010001111110(0 1)11(0 1)00(0 1 2)(0 1)1111(0 1)01(0 1)000----00-11000--000--------------0--------?--0-----0---0----------------0----------------------------0--------------------??--------------------0-0--0--0

Lobata 0100111111111000101011-100-0-000000-00000010000000-0--000000000000001100111011000111111110110000100(0 1)001111011111(0 1)(0 1)22111111101100----00-11000--000--------------0--------?--0-----0---0----------------0----------------------------0--------------------??--------------------0-0--0--0

Beroida 0100111111111000101001-100-0-000000-00000010000000-0--00000000000000110-10101100011111110011000101001111111101-1112000000----000----00-11000--000--------------0--------?--0-----0---0----------------0----------------------------0--------------------??--------------------0-0--0--0

Platyctenida 0100111111111000101011-100-0-000000-00000010000000-0--000000000000001111111011000(0 1)111111?01100000010001111110111(0 1)011(0 1)11111011010----00-11000--000--------------0--------?--0-----0---0----------------0----------------------------0--------------------??--------------------0-0--0--0

Cestida 0100111111111000101011-100-0-000000-00000010000000-0--000000000000001100111011000111111110110000000000111?0111110(0 1)22111111111000----00-11000--000--------------0--------?--0-----0---0----------------0----------------------------0--------------------??--------------------0-0--0--0

Ganeshida 0100111111111000101011-100-0-000000-00000010000000-0--000000000000001100111011000111111100110000100000111?0111110(0 1)2211??11101000----00-11000--000--------------0--------?--0-----0---0----------------0----------------------------0--------------------??--------------------0-0--0--0

Thalassocalycida 0100111111111000101011-100-0-000000-00000010000000-0--000000000000001100111011000111111100110000020000111?0111110(0 1)2211??11101000----00-11000--000--------------0--------?--0-----0---0----------------0----------------------------0--------------------??--------------------0-0--0--0

Bryozoa 0110111111110100111100-11100-00000000000000001000000010000000000001100--0001(0 1)--------0----00-0000-0000000000?0-0-0-0-0000----000----00--1010--000--------------0--------?--0-----0---0----------------0----------------------------0--------------------??--------------------0-0--0--0

Brachiopoda 0110111111110100111100-(0 1)11(0 1)0-00000000000000001000000010000000000001100--00010--------0----00-0000-0000000000?0-0-0-0-0000----000----00--1010--000--------------0--------?--0-----0---0----------------0----------------------------0--------------------??--------------------0-0--0--0

Phoronida 0110111111110100111100-11100-00000000000000000000000010000000000001100--00010--------0----00-0000-0000000000?0-0-0-0-0000----000----00--1010--000--------------0--------?--0-----0---0----------------0----------------------------0--------------------??--------------------0-0--0--0

Mollusca 0110111111110100111100-101(0 1 2)(0 1)000000000100001101010000000000000000001100--0000(0 1)--------0----00-1000-0000000000?0-0-0-0-0000----000----00--1010--000--------------0--------?--0-----0---0----------------0----------------------------0--------------------??--------------------0-0--0--0

Annelida 0110111111110100111100-101(0 1 2)0-0000000110000(0 1)101100000000000000000001100--00000--------0----00-1000-0000000000?0-0-0-0-0000----000----00--1010--000--------------0--------?--0-----0---0----------------0----------------------------0--------------------??--------------------0-0--0--0

Echinodermata 0110011111110100111000-10110-00000000000000000000011111000111000001100--0000(0 1)--------0----00-1(0 1)00-0000000000?0-0-0-0-0000----000----00--1010--000--------------0--------?--0-----0---0----------------0----------------------------0--------------------??--------------------0-0--0--0

Pterobranchia 0110111111110100111000-11110-00000000100000000000011111101101000001100--00000--------0----00-1000-0000000000?0-0-0-0-0000----000----00--1010--000--------------0--------?--0-----0---0----------------0----------------------------0--------------------??--------------------0-0--0--0

Chaetognatha 0110111111110100111000-10110-0000000010000-000000110010000000000001100--00000--------0----00-1000-0000000000?0-0-0-0-0000----000----00--1000--000--------------0--------?--0-----0---0----------------0----------------------------0--------------------??--------------------0-0--0--0

Onychophora 0110111111110100111000-1012111011011110001-010000000000000000000001100--00000--------0----00-1000-0000000000?0-0-0-0-0000----000----00--1000--000--------------0--------?--0-----0---0----------------0----------------------------0--------------------??--------------------0-0--0--0

Arthropoda 0110111111110100111000-101(0 1)1111000(0 1)1110001-010000000000000000000001100--00000--------0----00-1000-0000000000?0-0-0-0-0000----000----00--10{0 1}0--000--------------0--------?--0-----0---0----------------0----------------------------0--------------------??--------------------0-0--0--0

Nemertea 0110111111110100111100-101(0 1)0-0000000010000-100001000000000000000001100--00000--------0----00-1000-0000000000?0-0-0-0-0000----000----00--1010--000--------------0--------?--0-----0---0----------------0----------------------------0--------------------??--------------------0-0--0--0

Enteropneusta 0110111111110100111000-10110-0000000010000-000000011111101101000001100--00000--------0----00-1000-0000000000?0-0-0-0-0000----000----00--1010--000--------------0--------?--0-----0---0----------------0----------------------------0--------------------??--------------------0-0--0--0

Urochordata 0111111111110100111000-10110-0000000000000-000000010101110000100111100--00000--------0----00-1000-0000000000?0-0-0-0-0000----000----00--1010--000--------------0--------?--0-----0---0----------------0----------------------------0--------------------??--------------------0-0--0--0

Cephalochordata 0110111111110100111000-10110-0000000000000-000000011101110000110001100--00000--------0----00-1000-0000000000?0-0-0-0-0000----000----00--1010--000--------------0--------?--0-----0---0----------------0----------------------------0--------------------??--------------------0-0--0--0

Vertebrata 0101111111110100111000-10110-0000000000000-00000001(0 1)101110000111111100--00000--------0----00-1000-0000000000?0-0-0-0-0000----000----00--1000--000--------------0--------?--0-----0---0----------------0----------------------------0--------------------??--------------------0-0--0--0

Entoprocta 0110111111110100111100-111010000000-0000000100000000--0000000000001100--00001--------0----00-1100-0000000000?0-0-0-0-0000----000----00--1010--000--------------0--------?--0-----0---0----------------0----------------------------0--------------------??--------------------0-0--0--0

Nematoda 0110111111110100111000-101010100000-011101-000000000--0000000000001100--00000--------0----00-1000-0000000000?0-0-0-0-0000----000----00--1000--000--------------0--------?--0-----0---0----------------0----------------------------0--------------------??--------------------0-0--0--0

Nematomorpha 0110111111110100111000-101110100000-010101-000000000--0000000000001100--00000--------0----00-1000-0000000000?0-0-0-0-0000----000----00--1010--000--------------0--------?--0-----0---0----------------0----------------------------0--------------------??--------------------0-0--0--0

Priapulida 0110111111110100111000-101111100000-011111-000000000--0000000000001100--00000--------0----00-1000-0000000000?0-0-0-0-0000----000----00--1010--000--------------0--------?--0-----0---0----------------0----------------------------0--------------------??--------------------0-0--0--0

Gastrotricha 0110111111110100111100-10100-000000-010000-000000000--0000000000001100--00000--------0----00-1000-0000000000?0-0-0-0-0000----000----00--1000--000--------------0--------?--0-----0---0----------------0----------------------------0--------------------??--------------------0-0--0--0

Tardigrada 0110111111110100111000-1010111010101?10001-000000000000000000000001100--00000--------0----00-1000-0000000000?0-0-0-0-0000----000----00--1000--000--------------0--------?--0-----0---0----------------0----------------------------0--------------------??--------------------0-0--0--0

Kinorhyncha 0110111111110100111000-101?10100000-111111-000000000--0000000000001100--00000--------0----00-1000-0000000000?0-0-0-0-0000----000----00--1000--000--------------0--------?--0-----0---0----------------0----------------------------0--------------------??--------------------0-0--0--0

Loricifera 0110111111110100111000-101?10100000-011111-000000000--0000000000001100--00000--------0----00-1000-0000000000?0-0-0-0-0000----000----00--1010--000--------------0--------?--0-----0---0----------------0----------------------------0--------------------??--------------------0-0--0--0

Gnathifera 0110111111110100111100-10100-000000-010000-000000100--0000000000001100--00000--------0----00-1000-0000000000?0-0-0-0-0000----000----00--1000--000--------------0--------?--0-----0---0----------------0----------------------------0--------------------??--------------------0-0--0--?

Xenacoelomorpha 0100?1?1(0 1)1111111111000-0-0-0-000000-0?0000-0000000-0--0000000000001100--00000--------0----00-1000-0000000000?0-0-0-0-0000----000----00--1000--000--------------0--------?--0-----0---0----------------0----------------------------0--------------------??--------------------0-0--0--0

Platyhelminthes 0110111111110100111100-0-000-000000-010000-100000000--0000000000001100--00000-------?0----00-1000-0000000000?0-0-0-0-0000----000----00--1010--000--------------0--------?--0-----0---0----------------0----------------------------0--------------------??--------------------0-0--0--0

sphenothallus ??????????????????????????????00?0????????????????????????????????000?0???????????????????????0????????????????????????????????0??????????1????????????????????1??0????1?????????11??11??????????????????10?????????????????0?????????????????????????????????????????????????????????0

Conulariida ???????????????????????00?-???00?0?????????0??????????????????????003?0????????????????????0??0????????????????????????????????0-??????????????????????????????11101???0?--0-----10-?111?????????????????1????????????????????????2??????????????1??????????1?????????????????????????2

Olivoiides ???????????????????????00?-???00?0?????????0??????????????????????004?0????0???????????????0??0????????????????????????????????0-?????????0????????????????????1?10??????--0-----10-?11??????????????????11???????????????????1??1?1??????????????????????????????????????????????????2

Quadrapyrgites ???????????????????????00?-???00?0?????????0??????????????????????003?0????0???????????????0??0????????????????????????????????0-?????????0????????????????????1?10??????--0-----10-?11??????????????????11??????????????????????1????????????????????????????????????????????????????2

Pseudooides ???????????????????????00?-???00?0?????????0??????????????????????001?0????0???????????????0??0????????????????????????????????0-?????????0????????????????????1?10??????--0-----10-?????????????????????11???????????????????????????????????????????????????????????????????????????2

Nematostella 0110011111101100101001-0-0-0-000000-00000010000000-0--00000000000000000-10001010000--0----00-1000-0000000000?0-0-0-0-0000----000----00-11011110110-0001000000100-------01--11011010-111-101002111400001110-100203001?000110001000010--------------------??-------------------00-0--0-02

Anemonia 0110011111101100101001-0-0-0-000000-00000010000000-0--00000000000000500-10001010000--0----00-1000-0000000000?0-0-0-0-0000----000----00-11011110110-0001001000100-------01--11011010-111-10010211140000111?-1002051011001110111000010--------------------??-------------------10-0--0-02

Aiptasia 0110011111101100101001-0-0-0-000000-00000010000000-0--00000000000000500-10001010000--0----00-1000-0000000000?0-0-0-0-0000----000----00-11011110110-0001000000100-------01--11011010-111-100102111400101110-10020501??101110011000010--------------------??-------------------10-0--0-02

Metridium 0110011111101100101001-0-0-0-000000-00000010000000-0--00000000000000500-10001010000--0----00-1000-0000000000?0-0-0-0-0000----000----00-11011110110-0001000000100-------01--11011010-111-100102111400101110-1112050101101110011000010--------------------??-------------------00-0--0-02

Antipathes 0110011111101100101001-0-0-0-000000-00000010000000-0--00000000000000500-10001010000--0----00-1000-0000000000?0-0-0-0-0000----000----00-11011110110-0001001000100-------0100110110110011-100001011300001120-00010500??0?0000000000010--------------------??-------------------?100--0-02

Ceriantharia 0110?11111101100101001-0-0-0-000000-00000010000000-0--00000000000000000-10001010000--0----00-1000-0000000000?0-0-0-0-0000----000----000110111??????0001001000110-------01--11011010-111-10?002010201001010-000205001?010000001000000--------------------??-------------------?0-0--0-02

Corynactis 0110011111101100101001-0-0-0-000000-00000010000000-0--00000000000000500-10001010000--0----00-1000-0000000000?0-0-0-0-0000----000----00-11011110110-0001001000100-------01--11011010-111-10010210-400000-20-11021500??010000010000010--------------------??-------------------?0-0--0-02

Montastraea 0110011111101100101001-0-0-0-000000-00000010000000-0--00000000000000500-10001010000--0----00-1000-0000000000?0-0-0-0-0000----000----00-11011110110-0001001000100-------0100110110110111-10010210-400000-20-10021500??0?0000000000010--------------------??-------------------1110110-00

Porites 0110011111101100101001-0-0-0-000000-00000010000000-0--00000000000000500-10001010000--0----00-1000-0000000000?0-0-0-0-0000----000----00-11011110110-0001001000100-------0100110110110111-10010210-400000-20-10021300??0?0000000000010--------------------??-------------------1110110-00

Acropora 0110011111101100101001-0-0-0-000000-00000010000000-0--00000000000000500-10001010000--0----00-1000-0000000000?0-0-0-0-0000----000----00-11011110110-0001001000100-------0100110110110111-10010210-400000-20-10021200??0?0000000000010--------------------??-------------------1110110-00

Parazoanthus 0110011111101100101001-0-0-0-000000-00000010000000-0--00000000000000000-10001010000--0----00-1000-0000000000?0-0-0-0-0000----000----00-11011110110-0001001000100-------0100110110110111-101102211100001010-10020500??000011001000010--------------------??-------------------10-0--0-00

Anthomastus 0110011111101100101001-0-0-0-000000-00000010000000-0--00000000000000000-10001011000--0----00-1000-0000000000?0-0-0-0-0000----000----00-11011110110-0000001000000-------0100110110110011-11-000010000001000-00010100??0?0000001000010--------------------??-------------------?0-1--1102

Keratoisidinae 0110011111101100101001-0-0-0-000000-00000010000000-0--00000000000000000-10001011000--0----00-1000-0000000000?0-0-0-0-0000----000----00-11011110110-0000001000000-------0100110110110011-11-00001000000100?-00010100??0?0000001000010--------------------??-------------------?0-1--1102

Nephthyigorgia 0110011111101100101001-0-0-0-000000-00000010000000-0--00000000000000000-10001011000--0----00-1000-0000000000?0-0-0-0-0000----000----00-11011110110-0000001000000-------0100110110110011-11-000010000001000-00010100??0?0000001000010--------------------??-------------------?0-1--1112

Leptogorgia 0110011111101100101001-0-0-0-000000-00000010000000-0--00000000000000000-10001011000--0----00-1000-0000000000?0-0-0-0-0000----000----00-11011110110-0000001000000-------0100110110110011-11-000010000001000-00010100??0?0000001000010--------------------??-------------------?0-1--1112

Scleronepthya 0110011111101100101001-0-0-0-000000-00000010000000-0--00000000000000000-10001011000--0----00-1000-0000000000?0-0-0-0-0000----000----00-11011110110-0000001000000-------0100110110110011-11-000010000001000-00010100??0?0000001000010--------------------??-------------------?0-1--1102

Virgularia 0110011111101100101001-0-0-0-000000-00000010000000-0--00000000000000000-10001011000--0----00-1100-0000000000?0-0-0-0-0000----000----00-11011110110-0000001000000-------0100110110111011-11-000010000001000-00010100??0?0000001000010--------------------??-------------------00-1--0-02

Haliclystus 011001111110110010100100-0-0-000000-00000010000000-0--00000000000000100-10001010000--0----00-1-00-0000000000?0-0-0-0-0000----000----00-010111?10000010000100000100-011-12--10100010-01111--0------00010--000-000100--0?000000-0001210100-110001?-10000010-011000100000000000100-0--0002

Alatina 011001111110110010100110-0-0-000000-00000010000000-0--00000000000000100-10001010000--0----00-1-00-0000000000?0-0-0-0-0000----000----00-010111010001110100100000110-011-1?--110??010-01110--0------10010--000-000100--0?000000-0011210111010100101110010110021000110001000011000-0--0002

Chirodropida 011001111110110010100110-0-0-000000-00000010000000-0--00000000000000100-10001010000--0----00-1000-0000000000?0-0-0-0-0000----000----00-010111010001110100100000110-011-1?--110??010-01110--0------10010--000-000100--0?000000-0011210111010100101110010110021000110001000011100-0--0002

Atolla 011001111110110010100110-0-0-000000-00000010000000-0--00000000000000300-10001010000--0----00-1000-0000000000?0-0-0-0-0000----000----00-110111010001010000100000?????10???--?????0???01111--0------10000--??0-010500--0?000000-1001211100-10001101010001110000000111001000000000-0--0002

Nausithoe 011001111110110010100110-0-0-000000-00000010000000-0--00000000000000300-10001010000--0----00-1000-0000000000?0-0-0-0-0000----000----00-1101110100010100001000001110010000--1????010-01111--0------10000--100-010500--0?000000-1001211100-10001101010001110000000111001000000000-0--0002

Aurelia 011001111110110010100110-0-0-000000-00000010000000-0--00000000000000300-10001010000--0----00-1000-0000000000?0-0-0-0-0000----000----00-110111010001010000110000100-010111--1??11010-01101--0------10000--000-000300--0?000000-1001210100-10001101-11100110000000110001000000000-0--0002

Rhizostoma 011001111110110010100110-0-0-000000-00000010000000-0--00000000000000300-10001010000--0----00-1?00-0000000000?0-0-0-0-0000----000----00-110111010001010000110000100-010011--1??00010-01101--0------10000--000-000300--0?000000-1001210100-100010-1-1110011000001-11--01000-00-00-0--0002

Hydra 011011111110110010100110-0-0-000000-00000010000000-0--00000000000000300-10001010000--0----00-1100-0000000000?0-0-0-0-0000----000----00-0100100100011111001000000---00--13--0-----10-110----0------?0000--0-0-010?00--0?000000-000000----?----------??------------------------00-0--0002

Candelabrum 011011111110110010100110-0-0-000000-00000010000000-0--00000000000000300-10001010000--0----00-1000-0000000000?0-0-0-0-0000----000----00-010010010001111100100000101100--03000-----110110----0------?0000--000-000?00--0?000000-000000----?----------??------------------------00-0--0002

Hydractinia 011011111110110010100110-0-0-000000-00000010000000-0--00000000000000300-10001010000--0----00-1000-0000000000?0-0-0-0-0000----000----00-010110010001111100100000101100--0300???110111110----0------?0000--000-010300--0?000000-000001?????????????????????????????????????????00-0--0002

Ectopleura 011011111110110010100110-0-0-000000-00000010000000-0--00000000000000300-10001010000--0----00-1000-0000000000?0-0-0-0-0000----000----00-010010010001111100100000101100--03000-----1101101---0------?0000--000-010500--0?000000-00000100-110001011--1100000-10000011?001000000000-0--0002

Clytia 011011111110110010100110-0-0-000000-00000010000000-0--00000000000000300-10001010000--0----00-1100-0000000000?0-0-0-0-0000----000----00-010110010001110110100000101000--03001??1101111101---0------10000--000-010500--0?000000-00000100-1100010110-1100011100000011000?000000000-0--0002

Obelia 011011111110110010100110-0-0-000000-00000010000000-0--00000000000000300-10001010000--0----00-1100-0000000000?0-0-0-0-0000----000----00-010110010001110110100000101000--03001??1101111101---0------10000--000-010500--0?000000-00000100-1100010110-1100011100000011000?000000000-0--0002

Physalia 011011111110110010100110-0-0-000000-00000010000000-0--00000000000000300-10001010000--0----00-1000-0000000000?0-0-0-0-0000----000----00-010110010001111100101100102-00--0?011??1101111101---0------00000--0-0-0?0?00--0?000000-00000100-110001001--1100000-00000-11--00000-00-00-0--0002

Craseoa 011011111110110010100110-0-0-000000-00000010000000-0--00000000000000300-10001010000--0----00-1000-0000000000?0-0-0-0-0000----000----00-010110010001111100101100102-00--0?111??1101111101---0------00000--0-0-0?0?00--0?000000-00000100-110001001--1100000-00000-11--00000-00-00-0--0002

Abylopsis 011011111110110010100110-0-0-000000-00000010000000-0--00000000000000300-10001010000--0----00-1000-0000000000?0-0-0-0-0000----000----00-010110010001111100101100102-00--0?111??1101111101---0------00000--0-0-0?0?00--0?000000-00000100-110001001--1100000-00000-11--00000-00-00-0--0002

Agalma 011011111110110010100110-0-0-000000-00000010000000-0--00000000000000300-10001010000--0----00-1000-0000000000?0-0-0-0-0000----000----00-010110010001111100101100102-00--0?101??1101111101---0------00000--0-0-0?0?00--0?000000-00000100-110001001--1100000-00000-11--00000-00-00-0--0002

Nanomia 011011111110110010100110-0-0-000000-00000010000000-0--00000000000000300-10001010000--0----00-1000-0000000000?0-0-0-0-0000----000----00-010110010001111100101100102-00--0?101??1101111101---0------00000--0-0-0?0?00--0?000000-00000100-110001001--1100000-00000-11--00000-00-00-0--0002

Aeginia 011011111110110010100110-0-0-000000-00000010000000-0--00000000000000300-10001010000--0----00-1000-0000000000?0-0-0-0-0000----000----00-0101100100010000011000000-------03001??00110-0101---0-------0000--0-0-010?00--0?000000-0100-100-??0001110?-010000100000010?1010100000000-0--0002

Halitrephes 011011111110110010100110-0-0-000000-00000010000000-0--00000000000000300-10001010000--0----00-1000-0000000000?0-0-0-0-0000----000----00-0101100100011100001000000-------03001??00000-0101---0-------0000--0-0-010?00--0?000000-0100-100-1100010100-11000110000100111000000000000-0--0002

;

END;
