# Supplementary material for: A late-surviving stem-ctenophore from the Late Devonian of Miguasha (Canada)
Source: Sci Rep. 2021 Sep 24;11:19039. doi: 10.1038/s41598-021-98362-5 (PMC8463547; doi:10.1038/s41598-021-98362-5)
Supplement: Supplementary file 16 — Supplementary Figures. [file 41598_2021_98362_MOESM16_ESM.docx]

Fig. S1: Parsimony Analysis of 91-taxon dataset (3 contentious fossils deleted). (A) Strict and (B) majority-rule consensus of all retrieved most-parsimonious trees (481 000 trees, length 427). Numbers at nodes in B = percentage frequency.

Fig. S2: Parsimony Analysis of 94-taxon dataset. (A) Strict and (B) Majority-rule consensus of all retrieved most-parsimonious trees (343 000 trees, length 432). Numbers at nodes in B = percentage frequency.

Fig. S3: Parsimony bootstrap trees (majority-rule consensus) of 91-taxon and 94-taxon datasets, based on 200 nonparametric bootstrap replicates. Numbers at nodes = percentage frequency.

Fig. S4: Bayesian trees (majority-rule consensus) of 91-taxon and 94-taxon datasets. Numbers at nodes = posterior probability
